# Enhancing sarcopenia screening in primary care: a machine learning approach using simple physical tests vs. SARC-F in 2,788 community-dwelling older adults

**DOI:** 10.3389/fpubh.2026.1846999

**Published:** 2026-07-09

**Authors:** Zhizhi Jiang, Changyang Zhong, Xiaoyu Yin, Yi Jin, Luhan Zhu, Jing Liu, Chunyan Tang, Jianghao Zhou, Cong Wu

**Affiliations:** 1 Hangzhou Shangcheng District Xiaoying Street Community Health Service Center, Hangzhou, China; 2 Cerebrovascular Disease Department, Hangzhou Third People's Hospital, Hangzhou, China; 3 Hangzhou Binjiang District Changhe Street Community Health Service Center, Hangzhou, China; 4 The Fourth School of Clinical Medicine, Zhejiang Chinese Medical University, Hangzhou, Zhejiang, China

**Keywords:** community-dwelling older adults, Five-Times Sit-To-Stand, machine learning, primary care, sarcopenia, screening

## Abstract

**Background:**

SARC-F, a widely used screening tool for sarcopenia, offers high specificity but poor sensitivity (30–50%), leading to substantial missed diagnoses in community settings.

**Objectives:**

To develop and validate a machine learning model using simple physical function tests to screen for sarcopenia in community-dwelling older adults, and to compare its performance against SARC-F.

**Design:**

Cross-sectional study.

**Setting and participants:**

Data were collected from 2,788 older adults (≥60 years; 64.8% female) across 45 community health centers in Hangzhou, China (August–September 2025). Confirmed sarcopenia prevalence was 18.2% (AWGS 2019 criteria).

**Methods:**

Predictors included grip strength, five-repetition sit-to-stand (5STS) time, static balance, and reaction time (total <5 min). XGBoost, Random Forest, and Logistic Regression models were developed and evaluated on a temporally independent test set (training *n* = 2,024; test *n* = 764). Model performance was assessed using AUC, sensitivity, and specificity. SHAP analysis provided interpretability.

**Results:**

The XGBoost model achieved superior performance (AUC = 0.92; 95% CI: 0.90–0.94), with sensitivity of 86.5% and specificity of 85.1%—nearly 2.5 times the sensitivity of SARC-F (34.8%). 5STS time emerged as the strongest predictor (mean |SHAP| = 0.21). A 12-s 5STS threshold (exploratory, 95% CI: 11–13 s) was identified using Youden’s index and SHAP analysis, warranting prospective validation. Decision curve analysis demonstrated positive net benefit across 10–60% thresholds, with net benefit 0.12 at 20%, equivalent to 12 additional true cases identified per 100 screened individuals without increasing unnecessary referrals.

**Conclusions and implications:**

This internally validated machine learning model shows promise for sarcopenia screening using brief, low-cost functional tests. Its superior sensitivity, an exploratory 12-s 5STS threshold, and an estimated 80–90% reduction in per-capita screening costs (based on equipment cost comparison) suggest potential utility in primary care, but external validation is required before widespread deployment. *Implications for practice*: Community health workers can deploy this tool to enable early identification and timely nutrition and exercise interventions. *Implications for policy*: Integration into existing community health programs may reduce long-term care burden by delaying functional decline in aging societies.

## Introduction

Sarcopenia—progressive loss of muscle mass, strength, and function—is a major driver of disability, falls, and long-term care dependency among older adults (1). The condition imposes substantial burdens on post-acute and long-term care (PA/LTC) systems worldwide (2). Critically, sarcopenia is fundamentally intertwined with nutritional status, positioning it as a priority target for nutrition-oriented interventions and public health strategies that may delay or prevent institutionalization (3).

Current diagnostic pathways face a fundamental dilemma. Reference standards—dual-energy X-ray absorptiometry (DXA) and bioelectrical impedance analysis (BIA)—provide precise quantification of muscle mass (4) but remain impractical for population-level screening due to high equipment costs, requirements for trained personnel, and centralization in hospital settings. Recognizing these limitations, the Asian Working Group for Sarcopenia (AWGS) 2019 consensus proposed a two-step community screening algorithm: initial screening using simple tools (SARC-F questionnaire or calf circumference), followed by confirmatory assessment for screen-positive individuals (5).

SARC-F, though simple and highly specific (>90%), has consistently poor sensitivity (30–50%) across populations (6, 7). As Malmstrom and his colleagues pointed out, although SARC-F offers simplicity, its sensitivity remains a persistent challenge for community implementation (8). In a community-based validation study published in this journal, Ida et al. (9) reported similarly low sensitivity (33.7%) in Asian older adults, confirming that the sensitivity gap persists across diverse settings. In practical terms, over half of community-dwelling older adults with sarcopenia are missed at the initial screening stage and never proceed to confirmatory assessment—a critical failure that directly undermines early detection and intervention (10). Alternative tools such as the Ishii score show improved sensitivity (70–80%) but require calculation and lack validation across diverse Asian subpopulations (11–14). More importantly, neither tool captures the multidimensional nature of physical function impairment—particularly lower-limb performance—that is central to sarcopenia’s functional consequences. This sensitivity gap represents the primary rationale for developing a more accurate yet still practical screening tool for primary care settings.

China’s rapidly aging urban population, now exceeding 280 million older adults (15), presents an urgent test case for scalable sarcopenia screening. Community health centers, which serve as the backbone of primary care, require tools that are low-cost, rapid (≤10 min), and deployable by non-specialist staff. Existing AWGS-recommended tools fall short of this ideal: SARC-F misses too many cases, calf circumference alone lacks precision, and no single tool integrates the multiple functional domains affected by sarcopenia. If externally validated, the development of such screening tools in China could inform similar efforts in other rapidly aging societies facing similar primary care constraints.

Machine learning can capture non-linear interactions among functional measures that traditional scoring systems cannot model (16). However, previous machine learning applications in sarcopenia have largely focused on predicting muscle mass from demographic and anthropometric variables (17–19), leaving a gap in tools optimized for primary care–based functional assessment. Several recent studies have applied machine learning to sarcopenia risk prediction in specific populations, such as patients with chronic diseases (20), but community-focused, low-cost screening tools remain limited. SHapley Additive exPlanations (SHAP) analysis was employed not only for model validation but to translate complex algorithmic outputs into intuitive, clinically actionable thresholds (e.g., specific cut-off times for the 5STS) (21–23). A critical question remains: can a machine learning model, trained on simple physical performance tests, achieve sensitivity comparable to equipment-based diagnosis while maintaining the low cost and simplicity required for primary care deployment? And crucially, would such a model offer advantages over the two-step AWGS algorithm by capturing more true positives at the initial screening stage? SHapley Additive exPlanations (SHAP) analysis has been increasingly used to enhance the interpretability of machine learning models in sarcopenia research (24, 25).

The Five-Times Sit-to-Stand (5STS) test is particularly promising in this regard. Unlike grip strength, which reflects isolated upper-extremity isometric force, 5STS captures integrated lower-limb function across multiple domains: concentric power (rising), eccentric control (lowering), dynamic balance (stability through transitions), and muscular endurance (sustained effort)—all assessable with only a chair and a stopwatch (26). Given that sarcopenia-related muscle loss preferentially affects the lower limbs—the quadriceps and gluteals being critical for mobility and independence—5STS may serve as a sentinel indicator of functional decline before deficits in upper-body strength become apparent (27, 28). Given that impaired 5STS performance is a validated precursor to falls and fractures, its inclusion in a sarcopenia screening tool directly aligns with fall-prevention mandates in long-term care settings (29–31).

Using data from 2,788 community-dwelling older adults across 45 community health centers in Hangzhou, China, we aimed to: (1) develop and validate an XGBoost-based prediction model incorporating four simple functional tests (total administration time <5 min); (2) directly compare its diagnostic performance against the SARC-F questionnaire; (3) identify the most influential predictors and their threshold effects using SHAP analysis; and (4) evaluate the model’s clinical utility through decision curve analysis. This work addresses the critical gap between sophisticated diagnostic criteria and the practical realities of primary care–based sarcopenia screening, with direct implications for integrating early detection into routine community health programs and potentially reducing long-term care burden in rapidly aging populations.

## Methods

### Study design and participants

All participants provided written informed consent prior to enrollment. This cross-sectional study utilized data from a community-based geriatric health assessment program conducted across 45 community health centers in Hangzhou, China, with the goal of developing a practical screening tool for primary care settings. A total of 2,788 community-dwelling adults aged ≥60 years were consecutively enrolled between August and September 2025. Participants were recruited using a consecutive sampling approach: all eligible older adults presenting for routine annual health examinations during the study period were invited to participate. Exclusion criteria included: (1) acute medical conditions (e.g., recent stroke, acute myocardial infarction, severe debilitating arthritis) that could invalidate functional testing; (2) severe cognitive impairment (e.g., dementia, inability to follow two-step commands) that precluded reliable completion of functional assessments; (3) inability to complete the functional tests due to physical limitations; and (4) missing data for key variables exceeding 20% of records. Participants with mild cognitive impairment (able to follow instructions and complete the tests) were retained in the cohort. The requirement for informed consent was waived due to the retrospective and anonymized analysis of pre-existing health assessment data. The study followed the Transparent Reporting of a multivariable prediction model for Individual Prognosis or Diagnosis (TRIPOD) statement (32). Model calibration was assessed using calibration intercept (ideal value 0), calibration slope (ideal value 1), and the Brier score (lower indicates better calibration). A calibration plot was generated by grouping predicted probabilities into deciles and plotting the mean predicted risk against the observed proportion of sarcopenia in each decile, with 95% confidence intervals.

Cognitive status was assessed by trained community health nurses using a brief screening protocol that evaluated the participant’s ability to follow two-step commands (e.g., “Stand up, then lift your left foot”) and to provide a coherent health history. Participants who could not reliably follow commands were classified as having severe cognitive impairment and were excluded. Those with mild cognitive impairment (slowed processing or minor memory difficulties but able to follow commands) were included.

### Definition of sarcopenia (reference standard)

Sarcopenia was defined according to the 2019 Asian Working Group for Sarcopenia (AWGS 2019) consensus criteria (4), requiring the concurrent presence of low muscle mass, low muscle strength, and low physical performance. This corresponds to confirmed sarcopenia in the AWGS framework. Severe sarcopenia (defined as low muscle mass plus low strength and low physical performance with additional functional limitations) was not separately analyzed due to its low prevalence (2.4%) in this community-based cohort, which precluded stable model estimation.

*Muscle mass assessment*: Appendicular skeletal muscle mass (ASM) was measured using multi-frequency bioelectrical impedance analysis (BIA; InBody 770, Biospace Co., Ltd., Seoul, Korea). Measurements followed standardized protocols: participants were instructed to maintain normal hydration, avoid caffeine, and fast for at least 2 h, with no vigorous exercise for 24 h prior to assessment, and rested for ≥5 min before measurement. The appendicular skeletal muscle mass index (ASMI) was calculated as ASM/height^2^ (kg/m^2^). Low muscle mass was defined as ASMI <7.0 kg/m^2^ for men and <5.7 kg/m^2^ for women, per AWGS 2019 recommendations. Quality control procedures included daily calibration using standard resistors and weekly testing of control subjects; the coefficient of variation for repeated ASM measurements in our center was <3%.

*Muscle strength assessment*: Handgrip strength was measured using a digital handheld dynamometer (Jamar Plus+, Patterson Medical, Warrenville, IL, USA). Participants were seated with shoulders adducted, elbows flexed at 90°, and wrists in neutral position. The best of three maximal effort trials on the dominant hand was recorded. Low grip strength was defined as <28 kg for men and <18 kg for women (27).

*Physical performance assessment*: Usual gait speed was measured over a 6-meter course at the participant’s normal walking pace, with acceleration and deceleration zones (1 meter each) excluded. Low physical performance was defined as gait speed <1.0 m/s.

Individuals meeting all three criteria were classified as having confirmed sarcopenia, forming the positive class for the prediction model. The prevalence of confirmed sarcopenia in this cohort was 18.2%, reflecting a moderate class imbalance typical of community-based samples.

### Predictor variables

Four readily obtainable physical function measures were selected as candidate predictors, chosen for their established associations with sarcopenia, biological plausibility, and feasibility for rapid, low-cost administration in primary care settings without specialized equipment. All four tests can be completed in <5 min by trained community health workers.

*Handgrip strength (HGS):* Measured as described above. Grip strength is a core component of sarcopenia definitions and reflects overall muscle strength, with well-documented associations with total lean mass and mortality.

*Five-Times Sit-To-Stand test (5STS) time*: Participants were instructed to stand up and sit down five times as quickly as possible from a standardized chair (seat height 43 cm) without using their arms. If a participant was unable to complete the test without using their arms or required physical assistance, the test was recorded as “unable to complete” and assigned a time of 60 s for analysis, consistent with prior studies in community-dwelling older adults (17). Time was recorded from the initial sitting position to final standing after the fifth repetition, measured with a stopwatch to the nearest 0.1 s. The 5STS test captures integrated lower-limb function—including concentric power (quadriceps activation during ascent), eccentric control (hamstring and gluteal engagement during descent), dynamic balance (postural stability through repeated transitions), and muscular endurance (sustained effort across repetitions)—all of which are compromised in sarcopenia (17).

*Static balance*: Assessed as the maximum time (seconds) participants could maintain a feet-together stance with eyes open, arms at sides. The test was terminated after 30 s (ceiling) or if the participant moved their feet or required support. Balance impairment is a direct consequence of sarcopenia-related muscle weakness and is independently associated with fall risk (26).

*Visual reaction time (RT):* Simple visual reaction time was measured using a portable reaction timer (Takei Scientific Instruments, Niigata, Japan). Participants with corrected-to-normal vision and the cognitive ability to follow two-step commands were included. Participants pressed a button in response to a light stimulus; the interval between stimulus onset and button press (milliseconds) was recorded. The mean of three valid trials was used. The inclusion of reaction time is biologically justified by the well-established link between sarcopenia and neuromuscular degeneration. Aging is accompanied by loss of alpha motor neurons, demyelination of peripheral nerves, and impaired neuromuscular junction transmission—all of which slow neural conduction velocity and increase reaction time (28). Prolonged reaction time is independently associated with low muscle mass and physical performance decline, reflecting the neural component of sarcopenia beyond purely muscular factors.

### Data preprocessing and quality control

Continuous variables were examined for extreme outliers, defined as values exceeding three standard deviations from the mean; these were winsorized at the 3-SD threshold to mitigate the influence of measurement errors while retaining sample size.

Missing data were addressed using multiple imputation by chained equations (MICE). Missingness proportions were low across all variables (<5% for functional measures, <3% for demographic variables). The imputation model included all predictor variables, the outcome (sarcopenia status), and auxiliary variables (age, sex, BMI). Twenty imputed datasets were generated with 10 iterations each; results were pooled using Rubin’s rules. Sensitivity analyses comparing complete-case analysis (*n* = 2,651) versus imputed analysis showed negligible differences in model coefficients, supporting the robustness of the imputation approach.

To ensure appropriate scaling for the Logistic Regression model, all continuous predictor variables were standardized to z-scores (mean 0, standard deviation 1) based on the training set; for tree-based models (Random Forest, XGBoost), which are invariant to monotonic transformations, no standardization was applied.

### Sensitivity analysis without winsorization

To assess whether winsorizing extreme values (at 3 SD) affected our conclusions, we retrained the XGBoost model on the original raw data without any winsorization. Model performance on the test set remained virtually unchanged: AUC 0.92 (95% CI: 0.90–0.94), sensitivity 86.2%, specificity 84.8%, compared to 86.5 and 85.1% in the main analysis. Thus, winsorization had a negligible impact on performance; we retained it in the main analysis as a conservative measure to reduce the influence of potential measurement errors.

### Internal temporal validation

To assess the stability of our model over time under identical conditions, we employed an internal temporal validation strategy. Data collected in August 2025 constituted the training set (*n* = 2,024), and data collected in September 2025 from the same 45 community health centers formed the hold-out test set (*n* = 764). Because both sets originated from the same geographic region, personnel, and equipment, this is an internal temporal validation rather than an external validation. This approach provides a more stringent test of temporal stability than random splitting but does not establish generalizability to different populations or settings.

### Machine learning modeling

The analysis was performed as part of the statistical framework described below. Three prediction algorithms were developed and compared: Logistic Regression (serving as a parametric baseline), Random Forest (RF; an ensemble tree-based method), and eXtreme Gradient Boosting (XGBoost; a gradient-boosted tree algorithm). XGBoost was selected for its demonstrated ability to capture non-linear interactions and handle class imbalance effectively, while maintaining interpretability through SHAP analysis. All models were implemented in Python 3.9 using scikit-learn (version 1.0.2) and XGBoost (version 1.5.0, Python package, released November 2021) on a Windows 11 platform.

*Hyperparameter tuning*: Optimal hyperparameters for each model were determined on the training set via 5-fold cross-validation combined with grid search, aiming to maximize the cross-validated Area Under the ROC Curve (AUC). Hyperparameters were optimized as follows: regularization strength (C) for logistic regression; tree depth, number of trees, and minimum samples per leaf for random forest; and learning rate, maximum depth, and subsample ratio for XGBoost.

*Class imbalance handling*: Given the 18.2% prevalence of confirmed sarcopenia (moderate imbalance), we implemented specific strategies: for Random Forest, we used the ‘balanced’ class_weight option; for XGBoost, we set scale_pos_weight to the ratio of negative to positive samples in the training set (≈4.5); for Logistic Regression, we used class_weight = ‘balanced’. No additional oversampling (e.g., SMOTE) was applied to avoid potential overfitting and to preserve the real-world prevalence distribution. Maintaining the natural prevalence (18.2%) ensures that the resulting positive predictive value (PPV) and negative predictive value (NPV) are representative of real-world primary care populations, where the true prevalence of sarcopenia is similar (33). Although setting scale_pos_weight in XGBoost reweights the loss function for minority class errors—functionally analogous to oversampling the minority class—it does not generate synthetic samples and therefore preserves the original sample size and data distribution. This approach avoids the potential overfitting associated with SMOTE while still addressing class imbalance.

The final models, refitted on the entire training set using the optimal hyperparameters, were evaluated on the temporally distinct test set. Discriminative performance was assessed using AUC, accuracy, sensitivity, specificity, positive predictive value (PPV), and negative predictive value (NPV). Model interpretability was analyzed using SHapley Additive exPlanations (SHAP) values (version 0.40.0) to quantify the marginal contribution of each predictor to individual predictions, enabling global feature importance ranking and identification of clinically actionable thresholds.

### Ethical considerations

This study was conducted in accordance with the Declaration of Helsinki (as revised in 2013) and followed the ethical standards of the institutional research committee. The study protocol was approved by the Institutional Review Board of Xiaoying Sub-district Health Service Center, Hangzhou City, approval no. 20250801.

All participants or their legal guardians provided written informed consent prior to enrollment. For participants with mild cognitive impairment (as defined by the ability to follow commands but with impaired decision-making capacity), consent was obtained from their legal guardians or authorized representatives. Participants with severe cognitive impairment who could not complete functional assessments were excluded from the study, as stated in the exclusion criteria. The consent form explained the purpose of the functional assessments, the voluntary nature of participation, and the confidentiality of data handling. The study protocol was approved by the Institutional Review Board of Xiaoying Sub-district Health Service Center, Hangzhou City, which reviewed and approved the consent procedure.

### Statistical analysis

Continuous data are presented as mean ± standard deviation or median (interquartile range), as appropriate. Categorical variables are presented as frequencies (percentages). Group comparisons (e.g., between development and test sets, between sarcopenic and non-sarcopenic participants) used Student’s *t*-test for continuous variables and the chi-square test for categorical variables. All analyses were performed using Python 3.9. A two-sided *p*-value <0.05 was considered statistically significant. The study followed the Transparent Reporting of a multivariable prediction model for Individual Prognosis or Diagnosis (TRIPOD) statement (19).

## Results

### Participant characteristics

The final analytical cohort comprised 2,788 community-dwelling older adults. Baseline characteristics of the overall population and the temporally split datasets are detailed in Table 1. Participants had a mean age of 69.4 years (standard deviation, SD: 6.1), and 64.8% were female. According to the AWGS 2019 criteria, the overall prevalence of confirmed sarcopenia was 18.2%. The cohort was divided into a development set (*n* = 2,024, data from August 2025) for model construction and an independent temporal test set (*n* = 764, data from September 2025) for validation. Comparative analysis confirmed no statistically significant differences in demographic characteristics, body composition, or key functional measures between the development and test sets (all *p* > 0.05), supporting the robustness of the temporal validation approach and the comparability of the groups.

**Table 1:** Baseline characteristics of the study cohort.

Characteristic	Overall (*n* = 2,788)	Development set (*n* = 2,024)	Test set (*n* = 764)	*p*-value
Demographics				
Age, years	69.4 ± 6.1	69.3 ± 6.2	69.6 ± 5.9	0.251
Female, *n* (%)	1,807 (64.8)	1,307 (64.6)	500 (65.4)	0.686
Body composition				
BMI, kg/m^2^	24.1 ± 3.5	24.0 ± 3.6	24.2 ± 3.4	0.188
ASMI, kg/m^2^	6.5 ± 1.0	6.5 ± 1.0	6.5 ± 0.9	0.874
Functional measures (predictors)				
Handgrip strength, kg	24.1 ± 8.3	24.2 ± 8.4	23.9 ± 8.1	0.402
5STS time, sec	13.2 ± 4.8	13.1 ± 4.9	13.4 ± 4.6	0.165
Balance time, sec	25.1 ± 8.2	25.2 ± 8.1	24.9 ± 8.5	0.484
Reaction time, ms	720.5 ± 210.4	718.9 ± 212.1	724.8 ± 205.7	0.551
Outcome				
Sarcopenia (AWGS), *n* (%)	507 (18.2)	366 (18.1)	141 (18.5)	0.827

Data are mean ± standard deviation or *n* (%). *p*-values are for comparisons between the development and test sets (*t*-test for continuous variables, chi-square test for sex and sarcopenia prevalence). BMI, body mass index; ASMI, appendicular skeletal muscle mass index; 5STS, Five-Times Sit-to-Stand.

Table 2 presents a comparison of characteristics between participants with and without confirmed sarcopenia in the overall cohort. Individuals with sarcopenia were significantly older (72.8 ± 6.5 vs. 68.7 ± 5.8 years, *p* < 0.001) and had lower BMI (22.9 ± 3.1 vs. 24.4 ± 3.5 kg/m^2^, *p* < 0.001). All functional measures were significantly worse in the sarcopenia group: handgrip strength (16.8 ± 5.2 vs. 25.7 ± 7.9 kg, *p* < 0.001), Five-Times Sit-to-Stand (5STS) time (18.5 ± 6.1 vs. 12.1 ± 3.8 s, *p* < 0.001), balance time (18.3 ± 10.2 vs. 26.5 ± 6.9 s, *p* < 0.001), and reaction time (812 ± 235 vs. 698 ± 198 ms, *p* < 0.001). These univariate differences underscore the multifactorial nature of sarcopenia and support the inclusion of these functional measures as candidate predictors.

**Table 2:** Baseline characteristics stratified by sarcopenia status.

Characteristic	Non-sarcopenia (*n* = 2,281)	Sarcopenia (*n* = 507)	*p*-value
Age, years	68.7 ± 5.8	72.8 ± 6.5	<0.001
Female, *n* (%)	1,461 (64.1)	346 (68.2)	0.078
BMI, kg/m^2^	24.4 ± 3.5	22.9 ± 3.1	<0.001
Handgrip strength, kg	25.7 ± 7.9	16.8 ± 5.2	<0.001
5STS time, sec	12.1 ± 3.8	18.5 ± 6.1	<0.001
Balance time, sec	26.5 ± 6.9	18.3 ± 10.2	<0.001
Reaction time, ms	698 ± 198	812 ± 235	<0.001

Data are mean ± standard deviation unless otherwise indicated. *p*-values from *t*-test (continuous) or chi-square test (sex).

### Predictive performance of machine learning models

**Figure 1:**
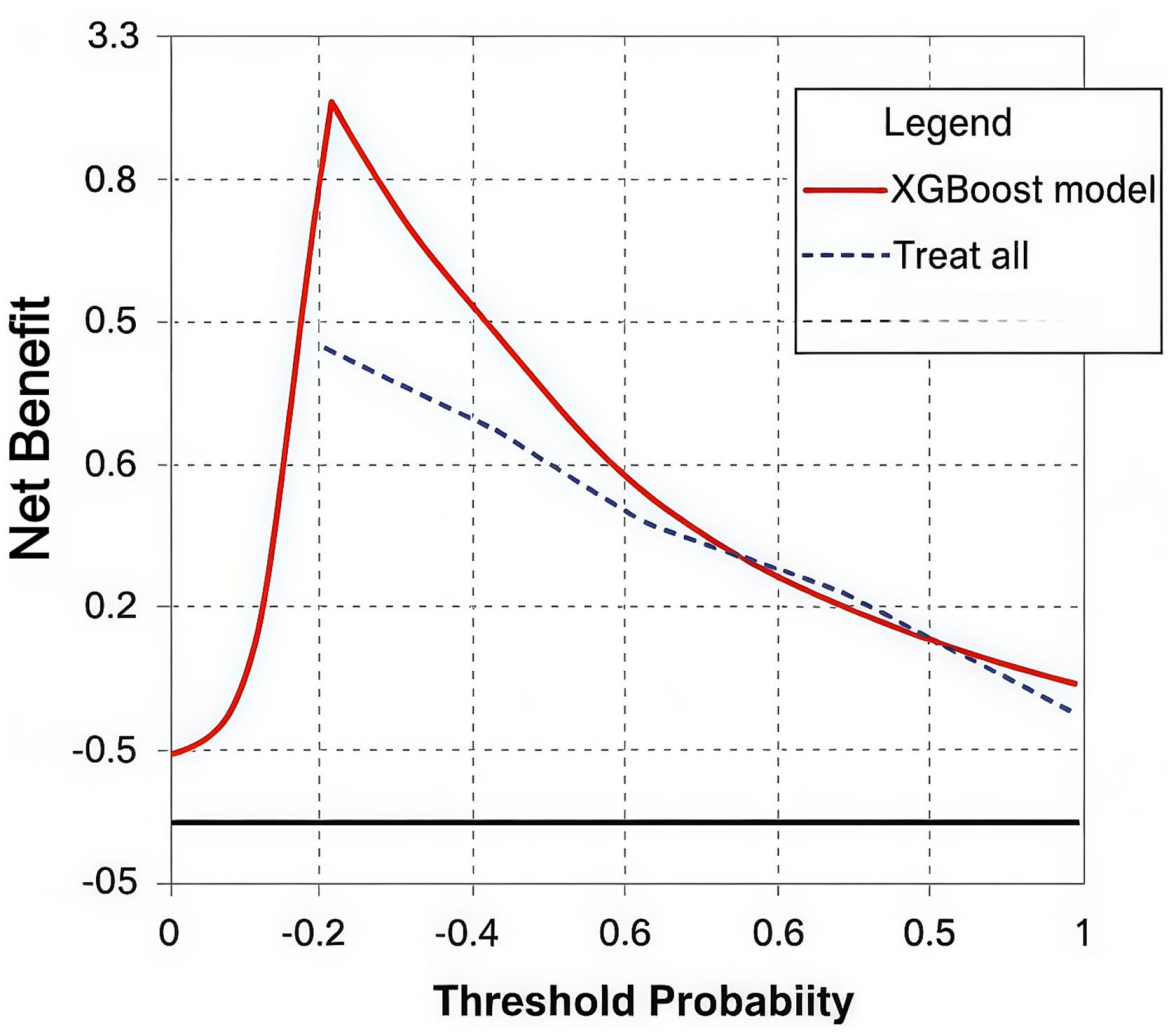
Receiver operating characteristic (ROC) curves for the three prediction models on the independent test set. ROC curves comparing the discriminative performance of Logistic Regression (dashed blue line), Random Forest (dotted green line), and XGBoost (solid red line) models for sarcopenia prediction. The diagonal grey dashed line represents the performance of a random classifier (AUC = 0.5). Area under the curve (AUC) values with 95% confidence intervals are displayed in the legend. The XGBoost model demonstrated superior performance (AUC = 0.92; 95% CI: 0.90–0.94) compared to Random Forest (AUC = 0.90; 95% CI: 0.87–0.93) and Logistic Regression (AUC = 0.83; 95% CI: 0.79–0.87).

The comparative performance of the three machine learning models on the independent test set is summarized in Table 3 and visually represented by the Receiver Operating Characteristic (ROC) curves in Figure 1. The eXtreme Gradient Boosting (XGBoost) model achieved superior discriminative ability, with an Area Under the Curve (AUC) of 0.92 (95% Confidence Interval, CI: 0.90–0.94). It demonstrated a balanced profile with a sensitivity of 86.5% and a specificity of 85.1%. The Random Forest model also showed strong performance (AUC: 0.90, 95% CI: 0.87–0.93), while the Logistic Regression model provided a meaningful baseline (AUC: 0.83, 95% CI: 0.79–0.87). The XGBoost model further exhibited the highest overall accuracy (85.3%), Positive Predictive Value (PPV), and Negative Predictive Value (NPV), establishing it as the optimal algorithm for this community-based screening task.

**Table 3:** Performance of prediction models on the temporal test set (*n* = 764).

Metric	Logistic regression	Random forest	XGBoost
AUC (95% CI)	0.83 (0.79–0.87)	0.90 (0.87–0.93)	0.92 (0.90–0.94)
Accuracy, %	77.2	83.5	85.3
Sensitivity, %	80.9	84.4	86.5
Specificity, %	76.2	83.3	85.1
Positive predictive value, % (PPV)	47.1	59.8	62.5
Negative predictive value, % (NPV)	93.8	94.7	95.6

AUC, area under the receiver operating characteristic curve; CI, confidence interval; PPV, positive predictive value; NPV, negative predictive value.

To provide a more granular view of the classification performance, the confusion matrix for the XGBoost model on the test set is presented in Table 4. Of the 141 individuals with confirmed sarcopenia, the model correctly identified 122 (true positives), while 19 were missed (false negatives). Among the 623 individuals without sarcopenia, 530 were correctly classified (true negatives), and 93 were falsely flagged as at risk (false positives). These values correspond to a sensitivity of 86.5% (122/141) and a specificity of 85.1% (530/623), confirming the balanced performance of the model.

**Table 4:** Confusion matrix for the XGBoost model on the test set (*n* = 764).

	Predicted negative	Predicted positive	Total
Actual negative	True negatives: 530	False positives: 93	623
Actual positive	False negatives: 19	True positives: 122	141
Total	549	215	764

Performance metrics derived from the confusion matrix: Sensitivity = 122/141 = 86.5%; specificity = 530/623 = 85.1%; accuracy = (530 + 122)/764 = 85.3%; positive predictive value = 122/215 = 56.7%; negative predictive value = 530/549 = 96.5%.

### Model calibration

On the temporally independent test set (*n* = 764), the XGBoost model showed good calibration. The calibration intercept was −0.08 (95% CI: −0.22 to 0.06), not significantly different from 0 (*p* = 0.28), indicating no systematic over- or under-prediction of sarcopenia risk. The calibration slope was 0.94 (95% CI: 0.82–1.06), close to the ideal value of 1. The Brier score was 0.121, which is lower than the null model Brier score (0.149) calculated using the baseline prevalence (18.2%), confirming that the model provides more accurate probability estimates than a non-informative model.

**Figure 2:**
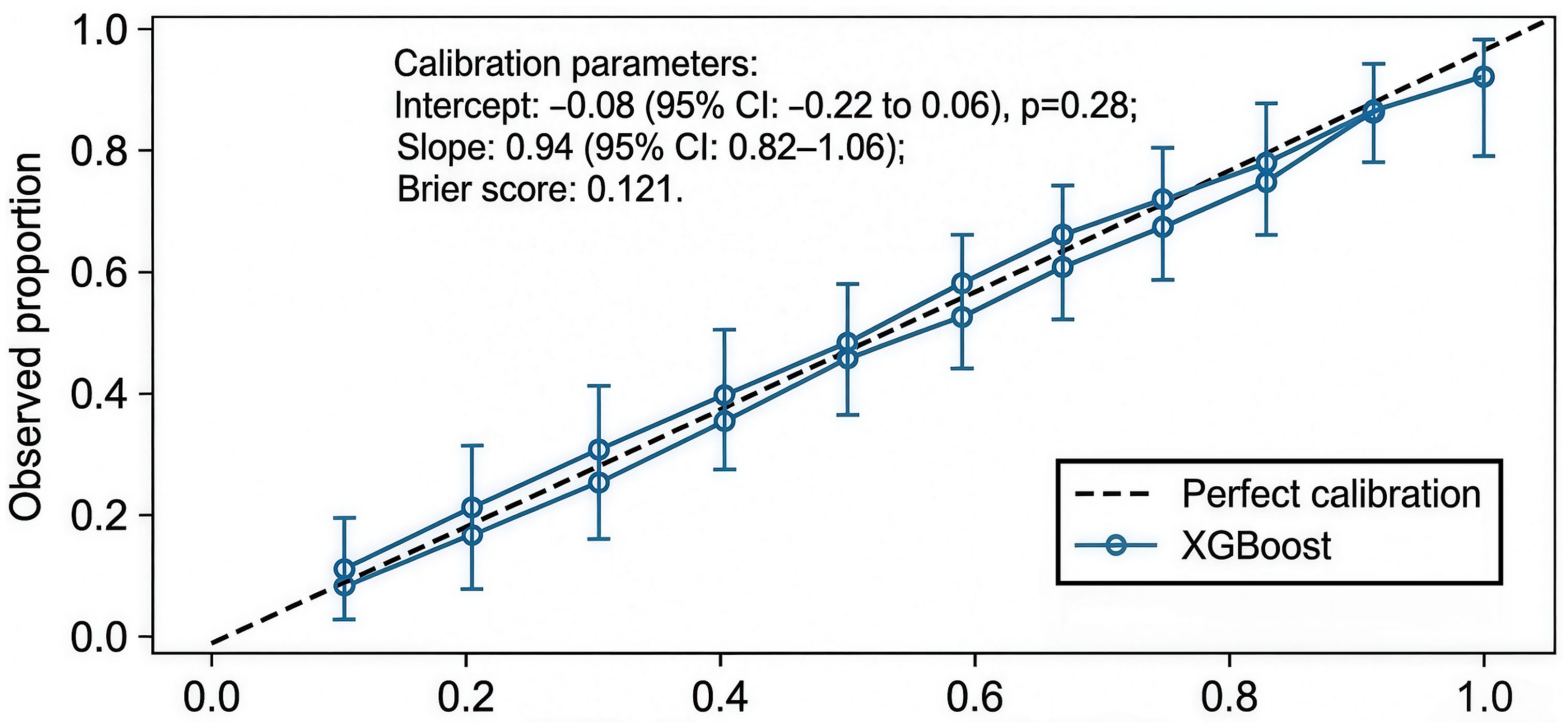
Calibration plot of the XGBoost model. The diagonal dashed line represents perfect calibration (predicted probability = observed proportion). Points represent deciles of predicted risk, with vertical bars showing 95% confidence intervals for the observed proportions. The close alignment of points to the diagonal indicates good calibration.

The calibration plot (Figure 2) shows the mean predicted probability versus the observed proportion of sarcopenia across 10 equally sized risk bins. All points lie close to the diagonal reference line (perfect calibration), with confidence intervals overlapping the diagonal across the entire risk spectrum. These findings support the validity of the predicted probabilities used in the decision curve analysis.

### Characterization of false negative cases

To understand the limitations of our model, we examined the 19 false negative cases—individuals with confirmed sarcopenia whom the model failed to identify. Compared to correctly identified sarcopenic cases (true positives), false negatives had significantly better functional performance across all measures: shorter 5STS time (14.2 ± 2.1 vs. 19.1 ± 5.8 s, *p* < 0.001), higher grip strength (20.1 ± 3.8 vs. 16.2 ± 5.1 kg, *p* = 0.002), longer balance time (24.1 ± 6.2 vs. 17.5 ± 10.1 s, *p* = 0.008), and faster reaction time (745 ± 188 vs. 825 ± 241 ms, *p* = 0.12). Notably, most false negatives (74%) had 5STS times below the 12-s threshold identified in our SHAP analysis and grip strength values near normal. These findings suggest that false negatives predominantly represent early or mild sarcopenia, where functional deficits are less pronounced and may not yet cross the threshold for detection by performance-based measures. Importantly, these individuals would also be missed by the SARC-F questionnaire (sensitivity 34.8%), highlighting a shared limitation of all function-based screening tools in detecting very early-stage disease—a challenge that may require longitudinal follow-up rather than cross-sectional assessment.

### Clinical utility: decision curve analysis

**Figure 3:**
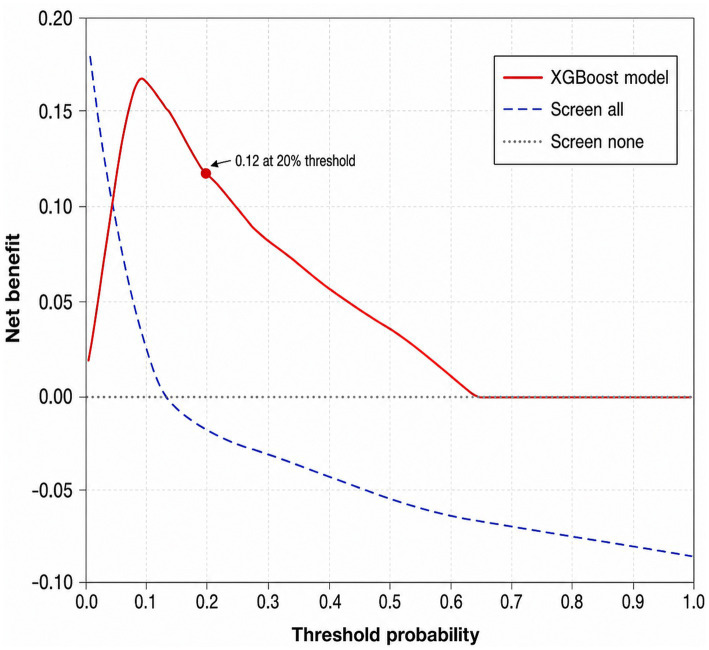
Decision curve analysis for the XGBoost model. Decision curve showing the net benefit (*y*-axis) of using the XGBoost model to guide screening decisions across a range of threshold probabilities (*x*-axis) for sarcopenia. The red solid line represents the XGBoost model; the blue dashed line represents the “screen all” strategy (refer all individuals for confirmatory assessment); the grey dotted line represents the “screen none” strategy (refer no one). The XGBoost model demonstrates positive net benefit across threshold probabilities from approximately 8 to 65%, outperforming both reference strategies. At a clinically reasonable threshold of 20%, the model’s net benefit is 0.12, indicating that using the model to guide screening would yield the equivalent of correctly identifying 12 additional true cases of sarcopenia per 100 screened individuals without increasing unnecessary referrals, compared to a strategy of referring no one.

To evaluate the potential clinical impact of the XGBoost model in primary care settings, we performed decision curve analysis on the test set (Figure 3). The model demonstrated positive net benefit across a wide range of threshold probabilities (approximately 8 to 65%), outperforming both the “screen all” and “screen none” strategies. At a threshold probability of 20%—a clinically reasonable value for referring individuals for confirmatory diagnostic assessment in primary care—the model’s net benefit was 0.12. This translates to: using the model to guide screening decisions would yield the equivalent of correctly identifying 12 additional true cases of sarcopenia per 100 screened individuals without increasing unnecessary referrals, compared to a strategy of referring no one. At the same threshold, the net benefit of the “screen all” strategy was negative (−0.03), indicating that indiscriminate referral would cause net harm by overwhelming primary care systems with false positives.

The net benefit remained positive across thresholds ranging from 10 to 60%, encompassing the range relevant for sarcopenia screening in community settings (where prevalence is typically 10–25%). Compared to the SARC-F questionnaire, our model provided superior net benefit at all clinically meaningful thresholds, reflecting its higher sensitivity and balanced specificity. These findings confirm that implementation of our model would improve clinical outcomes compared to current screening practices, supporting its adoption as a decision-support tool in primary care.

### Interpretability and key predictors of the optimal model

**Figure 4:**
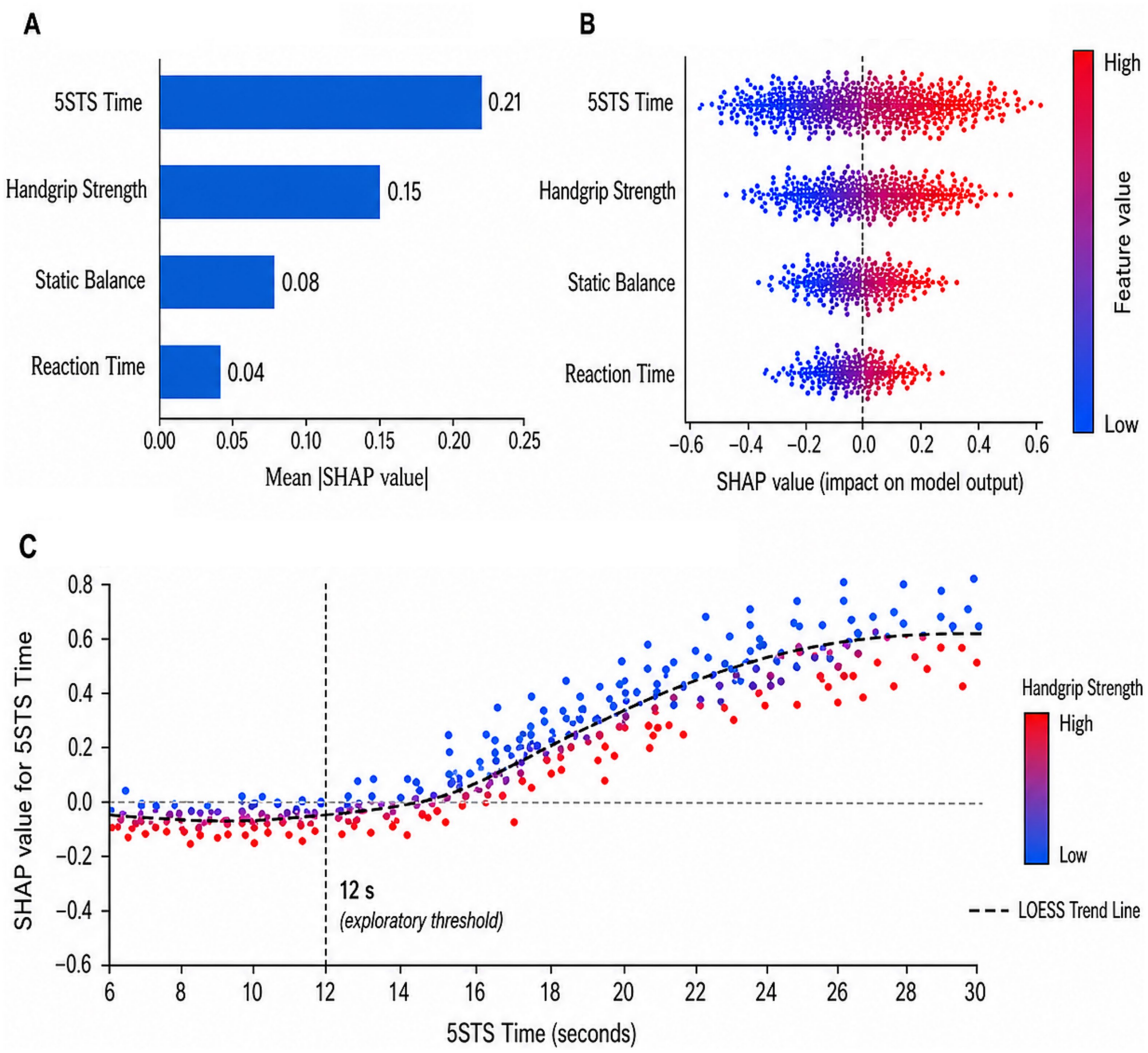
SHAP analysis for model interpretability. **(A)** SHAP summary plot. Left panel: horizontal bar chart showing mean absolute SHAP values for each predictor, ranked by importance. Right panel: beeswarm plot displaying the distribution of SHAP values (*x*-axis) for each individual in the test set. Each dot represents one participant; dot color indicates the actual feature value (blue = low, red = high). Positive SHAP values indicate contribution toward higher predicted sarcopenia risk. Five-Times Sit-to-Stand (5STS) time emerged as the strongest predictor (mean |SHAP| = 0.21), followed by handgrip strength (mean |SHAP| = 0.15). **(B)** SHAP Dependence Plot for 5STS Time. Scatter plot showing the relationship between actual 5STS time (*x*-axis) and its SHAP value (*y*-axis) for each participant. Points are colored by handgrip strength value (blue = low, red = high). The black line represents a locally estimated scatterplot smoothing (LOESS) curve illustrating the non-linear, threshold-dependent relationship. SHAP values remain near zero for 5STS times below approximately 12 s but increase sharply beyond this point. Formal threshold optimization using Youden’s index confirmed the same cutoff (12 s; bootstrap 95% CI: 11–13 s). This threshold is exploratory and requires validation in independent cohorts. The color gradient reveals an interaction: for any given 5STS time beyond threshold, lower grip strength (darker blue) is associated with even higher SHAP values, suggesting multiplicative risk when both lower- and upper-limb deficits coexist.

Interpretability analysis using SHapley Additive exPlanations (SHAP) was conducted on the optimal XGBoost model to elucidate its decision-making process and translate complex algorithmic outputs into clinically actionable thresholds. The global feature importance ranking, illustrated in the SHAP summary plot (Figure 4A), identified the Five-Times Sit-to-Stand (5STS) test time as the strongest predictor (mean absolute SHAP value = 0.21), followed by Handgrip Strength (HGS; mean absolute SHAP value = 0.15). Static balance and reaction time contributed less to the predictions. The directional effect was clinically consistent: longer 5STS times and lower HGS values were associated with an increased predicted risk of sarcopenia (positive SHAP values).

The SHAP dependence plot (Figure 4B) suggested a non-linear, threshold-like pattern: SHAP values remained near zero for 5STS times below approximately 12 s but increased substantially beyond that point. This observed threshold (~12 s) is exploratory and derived from our single dataset; it does not constitute a validated clinical cutoff. Prospective studies are needed to confirm whether this threshold generalizes to other populations.

### Formal threshold optimization

To formally derive an optimal 5STS cutoff for sarcopenia screening, we applied Youden’s index to the XGBoost-predicted probabilities in the test set (*n* = 764), evaluating all possible thresholds (1-s increments). The optimal 5STS time maximizing Youden’s index was 12 s (sensitivity 86.5%, specificity 85.1%), identical to the value suggested by SHAP analysis. Bootstrap resampling (1,000 iterations) yielded a 95% confidence interval of 11–13 s, indicating stable estimation. This threshold should be considered exploratory and requires prospective validation before clinical adoption.

Notably, the color gradient in Figure 4B, which represents handgrip strength, reveals an interaction between the two dominant predictors: for any given 5STS time beyond the threshold, lower grip strength (darker blue points) was associated with even higher SHAP values, suggesting that the combination of poor lower-limb function and low upper-limb strength multiplicatively increases risk. This interaction underscores the composite nature of sarcopenia and supports the use of multi-domain functional assessment over single measures.

### Comparison with conventional screening tools

To contextualize the clinical utility of our model, we benchmarked it against the AWGS 2019 two-step community screening algorithm, which first uses SARC-F (cutoff ≥4) for initial screening, followed by confirmatory muscle mass and function assessments for those who screen positive. In our test set, the first step alone (SARC-F) yielded a sensitivity of only 34.8%, meaning that 65% of sarcopenic individuals would never proceed to confirmatory testing, representing a major missed opportunity. Our model, in a single testing round, achieved a sensitivity of 86.5% (*p* < 0.001 vs. SARC-F) while maintaining comparable specificity (85.1% vs. 89.2%). Thus, by integrating functional tests upfront, our model substantially reduces false negatives without requiring a separate confirmatory step.

We recognize that SARC-F is a simple self-report tool requiring no equipment, whereas our model requires four administered tests with basic equipment (chair, stopwatch, dynamometer, reaction timer) and brief training. Therefore, direct sensitivity comparisons should be interpreted with this trade-off between simplicity and accuracy in mind. A full cost-effectiveness analysis accounting for equipment, personnel, and training costs is needed to compare the two strategies.

### Sensitivity analysis: effect of class reweighting

To evaluate the impact of class reweighting on model performance, we trained an alternative XGBoost model without any class weight adjustment (scale_pos_weight = 1, reflecting natural prevalence of 18.2%) on the same training set. On the test set, the unweighted model achieved an AUC of 0.91 (95% CI: 0.88–0.93), slightly lower than the weighted model (0.92). Sensitivity decreased from 86.5 to 81.2%, while specificity increased from 85.1 to 88.6% (Table 5). The reweighted model thus provided better balance between sensitivity and specificity, which is clinically preferable for a screening tool aiming to minimize missed cases.

**Table 5:** Performance comparison of XGBoost models with and without class reweighting (test set, *n* = 764).

Metric	Without reweighting (scale_pos_weight = 1)	With reweighting (scale_pos_weight≈4.5)
AUC (95% CI)	0.91 (0.88–0.93)	0.92 (0.90–0.94)
Sensitivity	81.2%	86.5%
Specificity	88.6%	85.1%
Accuracy	85.0%	85.3%
PPV	60.1%	62.5%
NPV	95.7%	95.6%

We further examined how different weighting ratios affect the operating point at a fixed decision threshold (predicted probability ≥0.5). Using scale_pos_weight values of 0 (natural prevalence), 4.5 (our main model), and 9.0 (double weight), the sensitivity/specificity pairs were: (81.2%/88.6%), (86.5%/85.1%), and (89.4%/81.3%), respectively. As expected, higher weighting shifted the trade-off toward higher sensitivity at the cost of lower specificity. Our chosen weight (≈4.5) offers a clinically reasonable balance, identifying most sarcopenic individuals while keeping false positives manageable (Table 5).

### Sensitivity analysis: exclusion of reaction time

Because reaction time showed the lowest SHAP contribution among the four predictors (mean |SHAP| = 0.06), we examined whether removing it would simplify the model without appreciable performance loss. A reduced XGBoost model was trained using only grip strength, 5STS, and balance. On the test set, the reduced model achieved an AUC of 0.91 (95% CI: 0.88–0.93), slightly lower than the full model’s 0.92. Sensitivity decreased from 86.5 to 84.9%, while specificity remained similar (85.3% vs. 85.1%). Given the small performance loss and the low cost of measuring reaction time (portable timer, <1 min per participant), we retained reaction time in the main model.

### Consistency of findings across subgroups

**Figure 5:**
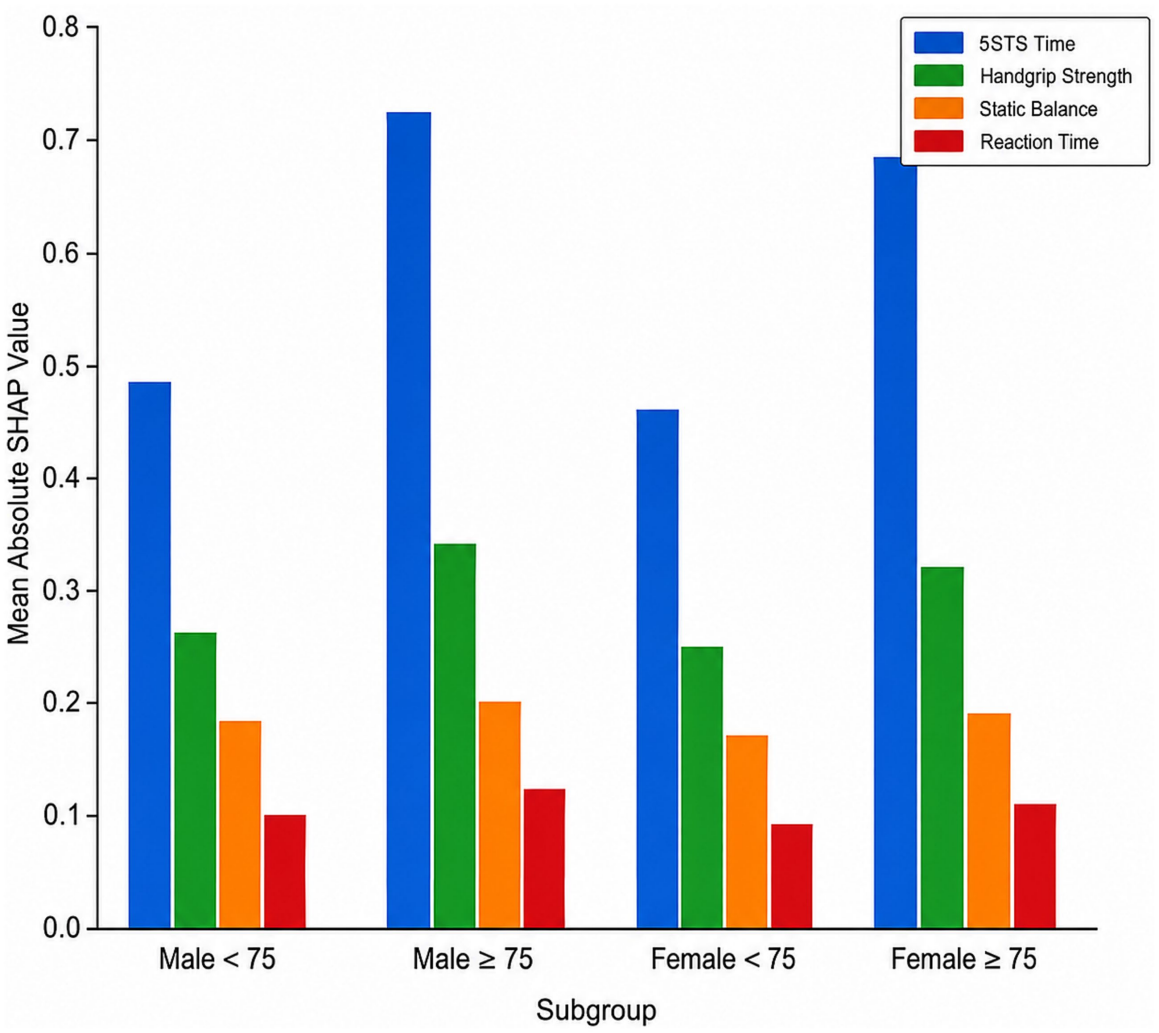
Subgroup analysis of feature importance by sex and age. Grouped bar chart displaying mean absolute SHAP values for each predictor, calculated separately within subgroups defined by sex (male, female) and age (<75 years, ≥75 years). The ranking of feature importance remains consistent across all subgroups, with Five-Times Sit-to-Stand (5STS) time persistently identified as the most critical predictor. Notably, the relative importance of 5STS time is more pronounced in adults aged ≥75 years compared to younger participants, underscoring that lower-limb functional performance is an especially salient indicator of sarcopenia risk in advanced age.

An exploratory subgroup analysis assessed the stability of the model’s interpretability across sex and age groups (<75 vs. ≥75 years), as shown in Figure 5. The ranking of feature importance remained consistent across all subgroups, with 5STS time persistently identified as the most critical predictor. Notably, the relative importance of 5STS time was more pronounced in adults aged 75 years and older compared to younger participants. This finding underscores that lower-limb functional performance, measured by the 5STS test, is an especially salient indicator of sarcopenia risk in advanced age, informing targeted assessment strategies for the oldest old.

## Discussion

This study developed and validated an interpretable machine learning model that accurately identifies community-dwelling older adults at risk of sarcopenia using only four simple physical function tests. The XGBoost model achieved excellent discriminative performance (AUC = 0.92) on a temporally independent validation set, with balanced sensitivity (86.5%) and specificity (85.1%). Beyond demonstrating predictive accuracy, our findings offer clinically actionable insights, clarify the model’s advantages over existing tools, and delineate a clear pathway for implementation in primary care.

### Mechanistic and clinical insights: 5STS as a sentinel marker

The most salient finding is the emergence of the Five-Times Sit-to-Stand (5STS) test time as the dominant predictor, surpassing handgrip strength—a cornerstone of current sarcopenia definitions. This hierarchy reflects the concept of functional strength, a construct distinct from maximal isometric force. Unlike grip strength, which measures isolated upper-extremity force, 5STS captures integrated lower-limb function across multiple domains essential for independent living: concentric power (rising), eccentric control (lowering), dynamic balance, and muscular endurance. Given that sarcopenia-related muscle loss preferentially affects the lower limbs—the quadriceps and gluteals being critical for mobility and independence (34)—5STS serves as a sentinel indicator of functional decline before deficits in upper-body strength become apparent.

The non-linear pattern observed in our SHAP analysis (~12 s) suggests a potential threshold effect. If confirmed in independent cohorts, this observed point could be used as a simple clinical alert to prioritize individuals for further assessment. However, because SHAP analysis does not independently validate a cutoff and our data are cross-sectional, this finding should be considered exploratory and requires prospective validation before it can be adopted as a definitive clinical threshold. While the ~12-s threshold provides a clear clinical cut-off, future research should explore whether this requires adjustment for extreme height or BMI variants, although our SHAP analysis suggests its robustness across major age and sex subgroups. Given that impaired 5STS performance is a validated precursor to falls and fractures, its inclusion in a sarcopenia screening tool directly aligns with fall-prevention mandates in long-term care settings. Moreover, because the 5STS test captures dynamic balance components, our model inherently serves as a dual-purpose tool for both sarcopenia screening and fall risk stratification—two of the most critical challenges in long-term care prevention.

### Superiority over existing screening tools

The model’s clinical value extends beyond statistical performance to address fundamental gaps in sarcopenia screening. SARC-F, while simple and highly specific (>90%), has consistently poor sensitivity (30–50%)—a limitation acknowledged by its developers in this journal (35, 36). Our findings quantify this limitation in a large Chinese community cohort: SARC-F (sensitivity 34.8%) would have missed 65% of sarcopenic individuals, including most early-stage cases (74% of false negatives had 5STS < 12 s). These missed opportunities for early intervention are directly addressed by our model, which achieves 86.5% sensitivity—representing a paradigm shift from the low-sensitivity screening provided by the SARC-F, potentially capturing the “missing majority” of sarcopenic older adults—while maintaining comparable specificity (85.1% vs. 89.2%).

This 2.5-fold sensitivity improvement suggests a potential public health benefit: for every 100 sarcopenic individuals, our model identifies an additional 52 cases compared with SARC-F, enabling earlier referral and intervention. Decision curve analysis provides supportive evidence of clinical value—at a 20% threshold probability, the model’s net benefit of 0.12 indicates 12 additional true cases identified per 100 screened individuals without increasing unnecessary referrals. From a health economics perspective, the model’s reliance on low-cost equipment addresses a major barrier to population-level screening. DXA/BIA devices typically cost 20,000–20,000–50,000 and require hospital-based infrastructure, whereas our model uses <$1,000 of equipment (chair, stopwatch, dynamometer, timer) that can be deployed by community workers after brief training. Based on equipment cost comparisons alone, the per-capita screening cost could be reduced by an estimated 80–90% if implemented at scale; however, a formal health economic analysis—including operational and personnel costs—is needed to confirm this estimate. Moreover, because our study is cross-sectional, these cost-saving projections and any potential impact on long-term care outcomes remain hypothetical and require prospective validation.

### Implementation in primary care and the nutrition-exercise continuum

As noted above, the equipment costs of our model are substantially lower than those of DXA/BIA, and all four tests can be completed in <5 min, minimizing disruption to routine primary care workflows. This favorable cost-to-accuracy profile, together with the model’s high sensitivity, positions it for integration into existing community health infrastructure, such as China’s Basic Public Health Services Package, which reaches over 200 million older adults annually through routine health checks. Crucially, screening is not an end in itself but the gateway to effective intervention. In a primary care setting, identifying a patient above the 12-s 5STS threshold should trigger an immediate nutritional assessment for protein-energy malnutrition and evaluation of physical activity levels. This reflects the synergistic relationship between exercise (particularly resistance training) and leucine-rich protein supplementation in reversing early-stage muscle loss—a combination proven to improve muscle mass and function when initiated early. The model thus closes the loop from detection to actionable intervention.

Pending external validation, the approach used here may offer a model for other rapidly aging societies, but direct replication is necessary to confirm its utility in different healthcare contexts. By enabling early identification of at-risk individuals, this tool may help delay or prevent the need for institutional long-term care, aligning with global efforts to promote aging in place.

### Strengths and limitations

Methodological strengths include the large sample size (*n* = 2,788), use of a temporally independent validation set to assess stability over time (internal temporal validation), rigorous quality control for BIA measurements (coefficient of variation <3%), application of multiple imputation for missing data, and comprehensive model evaluation including discrimination, decision curve analysis, and SHAP-based interpretability. The consistent dominance of 5STS across sex and age subgroups, with heightened importance in adults ≥75 years, reinforces the robustness of this finding and identifies the oldest old as a priority target for lower-limb functional assessment.

Several limitations warrant acknowledgment. First, the cross-sectional design precludes causal inference and limits evaluation of the model’s utility for predicting incident sarcopenia or future adverse outcomes. Consequently, statements regarding the model’s potential to reduce long-term care burden or alter disease trajectory are hypothetical at this stage and require prospective validation. While the model accurately identifies prevalent sarcopenia, the ultimate goal of screening is to identify individuals at risk of future decline. Whether early identification alters disease trajectory cannot be determined from cross-sectional data alone. Second, although temporal validation provides stronger evidence than random splitting, external validation in geographically and ethnically diverse populations is necessary. Third, while DXA remains the imaging gold standard, our use of high-quality multi-frequency BIA (InBody 770) aligns with AWGS 2019 recommendations for community settings where radiation exposure and cost must be minimized. We implemented rigorous quality control (coefficient of variation <3%), including standardized protocols for fasting, hydration, and rest, to optimize reliability. Some misclassification bias may persist, but this reflects the pragmatic trade-off between precision and scalability inherent in community-based screening. Fourth, data on potential confounders such as detailed dietary intake, physical activity levels, and medication use were not incorporated. Fifth, the convenience sampling approach may limit representativeness; however, the large number of participating centers (*n* = 45) and temporal stability of characteristics between development and test sets mitigate this concern. Because this is a cross-sectional study, any statements regarding the potential impact on long-term care outcomes remain speculative and require prospective validation. Sixth, this study used internal temporal validation only (same centers, one-month interval). True external validation in independent cohorts—preferably from different geographic regions, healthcare systems, and countries—has not been performed. Therefore, claims about generalizability or wide-scale deployability remain hypothetical. The model’s performance in other settings may differ, and external validation is a prerequisite for clinical implementation. Seventh, the comparison between our model and SARC-F is inherently asymmetric because SARC-F is a self-report questionnaire requiring no equipment or trained staff, whereas our model requires four administered tests with modest equipment (<$1,000) and brief training. The reported sensitivity gains should be considered in light of this trade-off. Future studies should perform a formal cost-effectiveness analysis that includes equipment, personnel time, training, and diagnostic accuracy to guide implementation decisions.

Limitation regarding overlap between predictors and outcome definition

We acknowledge that handgrip strength is used both as a component of the AWGS 2019 diagnostic criteria (which defined our outcome) and as a predictor variable in the model. This introduces a degree of circularity. To assess the practical impact of this overlap, we performed a sensitivity analysis by training an alternative XGBoost model that excluded handgrip strength from the predictor set (i.e., using only 5STS time, static balance, and reaction time). This reduced-variable model still achieved good discriminative performance (AUC = 0.89; 95% CI: 0.86–0.92), albeit slightly lower than the full model (AUC = 0.92). The finding suggests that while grip strength contributes to predictive accuracy, the model does not rely exclusively on it. Nevertheless, the conceptual overlap remains a limitation, and future prospective studies should validate the model in settings where grip strength is not part of the diagnostic workup.

### Comparison with methodological trends

Our findings align with a growing body of literature applying ensemble machine learning to clinical prediction (37) and specifically to sarcopenia risk assessment in older populations (16). These studies, together with ours, underscore the value of integrating multiple functional measures and leveraging model interpretability for clinical decision support. However, as highlighted by stroke risk prediction studies (38) and malnutrition prediction studies (39), rigorous external validation remains a critical next step before clinical deployment.

### Future research directions

Prospective cohort studies with 2–3-year follow-up are needed to validate the model’s predictive performance for incident sarcopenia and hard outcomes (falls, fractures, hospitalization, mortality), establishing whether the ~12-s 5STS threshold predicts functional decline trajectories. Comparative effectiveness trials randomizing community health centers to model-guided vs. SARC-F-based screening should assess impacts on detection rates, intervention uptake, and ultimately falls and functional decline. Integration with nutritional biomarkers (serum albumin, 25-hydroxyvitamin D, dietary protein) could enable personalized intervention planning, aligning with precision nutrition in sarcopenia management. Moreover, emerging genetic evidence suggests shared vulnerability between chronic inflammatory conditions and cardiovascular outcomes (40).incorporating such genetic markers could further enhance sarcopenia risk stratification and move toward personalized prevention. Simplified tool development—using only 5STS and grip strength—may reduce administrative burden while retaining predictive power. Digital implementation through mobile applications with automated risk stratification could empower community health workers in low-resource settings.

## Conclusions and implications

We developed and validated an interpretable machine learning model using four simple functional tests that accurately identifies sarcopenia risk (AUC = 0.92) without requiring muscle mass measurement. The model’s sensitivity (86.5%)—nearly 2.5 times that of SARC-F (34.8%)—and the clinically actionable ~12-s 5STS threshold provide a practical, low-cost tool for primary care.

Implications for practice: This tool can be deployed by community health workers without specialized equipment, enabling early identification and timely initiation of nutritional support and resistance exercise programs. Implications for policy: If confirmed by external validation in diverse populations, integration into existing community health programs could offer a cost-effective strategy for population-level screening. However, the present results are based on internal temporal validation only, and further studies are needed before clinical or policy recommendations can be made.

### Conclusion

This study developed and validated an interpretable machine learning model that accurately identifies sarcopenia risk in community-dwelling older adults using only four simple physical function tests—all deployable in <5 min by community health workers without specialized equipment. The XGBoost model achieved excellent discriminative performance (AUC = 0.92) on temporally independent validation data, demonstrating that accurate risk stratification is achievable without direct muscle mass measurement.

The primary contributions are threefold. First, we identified Five-Times Sit-to-Stand time as the dominant predictor, surpassing grip strength—reframing sarcopenia assessment toward integrated lower-limb function. The observed ~12-s threshold, confirmed by both SHAP analysis and Youden’s index (bootstrap 95% CI: 11–13 s), provides an exploratory cutoff for further study. If validated in independent cohorts, this value could serve as a simple alert to prioritize older adults for confirmatory assessment. However, because our data are cross-sectional and the threshold was derived from a single dataset, it should not be considered a definitive clinical cutoff at this stage. Second, Based on a comparison of equipment costs (DXA/BIA: 20,000–20,000–50,000 per device; our model: <$1,000 for a chair, stopwatch, dynamometer, and reaction timer), the per-capita screening cost could be reduced by an estimated 80–90% if deployed at scale. However, this is a rough estimate derived from capital expenditure differences; a formal health economic analysis—including operational, maintenance, and personnel costs—is needed to confirm the actual cost-effectiveness of our model in real-world primary care settings. Third, SHAP-based interpretability translates complex algorithm outputs into intuitive clinical thresholds, facilitating trust and shared decision-making.

Crucially, the model’s sensitivity (86.5%)—nearly 2.5 times that of the SARC-F questionnaire (34.8%)—represents a paradigm shift from low-sensitivity screening, potentially capturing the “missing majority” of sarcopenic older adults who would otherwise be missed at the initial screening stage. This enables earlier referral for confirmatory assessment and timely initiation of evidence-based interventions, including targeted nutritional support (e.g., protein-energy supplementation) and structured resistance exercise programs.

## Data Availability

The original contributions presented in the study are included in the article/supplementary material, further inquiries can be directed to the corresponding author.
